# Bold Colors in a Cryptic Lineage: Do Eastern Indigo Snakes Exhibit Color Dimorphism?

**DOI:** 10.1371/journal.pone.0064538

**Published:** 2013-05-15

**Authors:** Jennifer Deitloff, Valerie M. Johnson, Craig Guyer

**Affiliations:** 1 Department of Biological Sciences, Auburn University, Auburn, Alabama, United States of America; 2 Department of Entomology, Iowa State University, Ames, Iowa, United States of America; Arizona State University, United States of America

## Abstract

Many species exhibit variation in the color of their scales, feathers, or fur. Various forms of natural selection, such as mimicry, crypsis, and species recognition, as well as sexual selection, can influence the evolution of color. Eastern Indigo Snakes (*Drymarchon couperi*), a federally threatened species, have coloration on the sides of the head and the chin that can vary from black to red or cream. Despite significant conservations efforts for this species, little is known about its biology in the field. Past researchers have proposed that the color variation on the head and chin is associated with the sex of the individual. Alternatively, color might vary among individuals because it is controlled by genes that are under natural selection or neutral evolution. We tested these alternative hypotheses by examining whether coloration of the sublabial, submaxillary, and ventral scales of this species differed by sex or among clutches. We used color spectrometry to characterize important aspects of color in two ways: by examining overall color differences across the entire color spectrum and by comparing differences within the ultraviolet, yellow, and red colorbands. We found that Eastern Indigo Snakes do not exhibit sexual dichromatism, but their coloration does vary among clutches; therefore, the pattern of sexual selection leading to sexual dichromatism observed in many squamates does not appear to play a role in the evolution and maintenance of color variation in Eastern Indigo Snakes. We suggest that future studies should focus on determining whether color variation in these snakes is determined by maternal effects or genetic components and if color is influenced by natural selection or neutral evolutionary processes. Studying species that exhibit bright colors within lineages that are not known for such coloration will contribute greatly to our understanding of the evolutionary and ecological factors that drive these differences.

## Introduction

Animal color signals undergo selection through many different evolutionary processes that may be at odds with one another. For example, sexual selection may lead to bright conspicuous coloration, but natural selection via predator avoidance would favor cryptic colors. Sexual dichromatism (when males and females within a species differ in color traits) is a common result of sexual selection for color traits [1]. In many vertebrates, color is used to signal sexual identity and mate quality, and, thus, color is used to select mates [2]–[5]. However, possession of conspicuous color is often at odds with predator avoidance, implying that the benefits of producing colors that attract mates outweigh the survival costs of being more conspicuous to predators [6] or outweigh costs of producing color pigments or structures [7], [8]. A first step in understanding how color may be used by individuals is to determine the extent to which color correlates with sex identification or relatedness.

Studies of vertebrate groups in which color variation is rare may provide novel insights into the evolutionary development and adaptive significance of these traits. For example, among snakes, a diverse group that includes some 3000 species that are nested within the group Squamata [9], display color variation among individuals [10]–[19], but some aspects of color variation appears to be more restricted than it is among the nearly 6,000 species of lizards comprising the rest of the group Squamata. In particular, many groups of lizards are primarily visually-oriented, utilize color signals, and exhibit sexual dichromatism (i.e. agamids: [20]; anoles: [21], [22]; chameleons: [23]; collared lizards: [24]). This contrasts with snakes, which rarely exhibit sexual dichromatism and, in the best studied cases of this feature, do not support sexual selection as the driving force generating sexual dichromatism.

**Figure 1:**
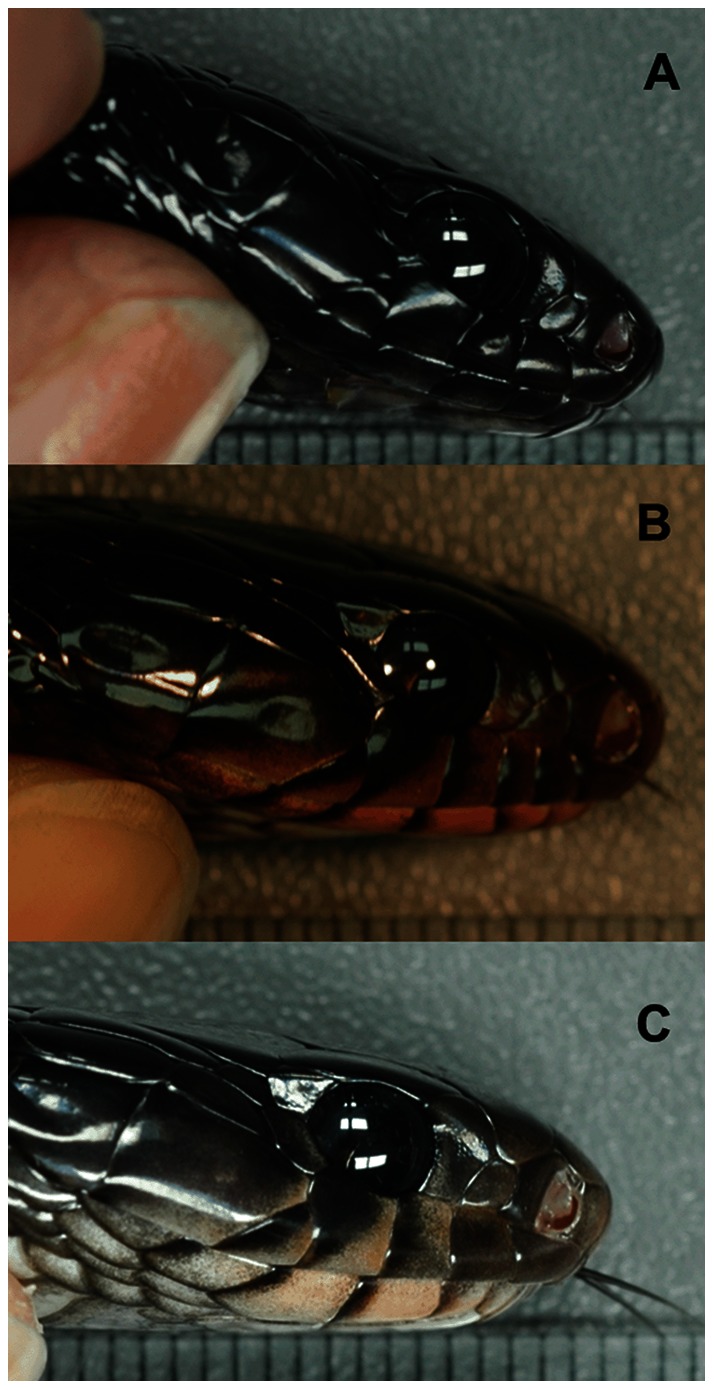
Images of three individuals displaying color variation in the sublabial scales. (a) Individual from clutch D with solid black sublabial scales. (b) Individual from clutch F with red sublabial scales. (c) Individual from clutch K with cream sublabial scales. The scale within each photograph represents mm.

The Eastern Indigo Snake (*Drymarchon couperi*), a large, mostly iridescent black serpent found in open pine habitats of the southeastern United States, may represent one of the rare snake species in which sexes differ in color pattern. This species traverses a variety of habitats during different seasons: during the active season, individuals move widely among upland sites and forage in hydric soils along creeks and during winter, they stay restricted to upland xeric sites with deep sandy soil [25]. The chin and lateral surfaces of the head as well as the first several ventral scales of these snakes vary in color from solid black to cream or red (Fig. 1). These features have been mentioned and praised by naturalists and pet enthusiasts for decades; however, no study has quantitatively analyzed the color variation among Eastern Indigo Snakes, perhaps due to their current rarity and protection under the U.S. Endangered Species Act. In general, snakes are thought to be derived from a nocturnal ancestor that lost colored oil droplets on the photoreceptors and in which cones transmuted to rods [26]. Anatomy of the eyes of derived, diurnal snakes, like Eastern Indigo Snakes, suggest a tertiary transmutation of rods to cones [26], a feature that appears to create limited color vision [27], [28].

Mount [29] speculated that individual Eastern Indigo Snakes with bright red or cream along the lower edge of the mouth tend to be females; in contrast, Moulis [30] speculated that bright red individuals are males. However, neither author, nor others, has quantitatively examined sexual dichromatism in this species. Because Eastern Indigo Snakes are part of a radiation of North American racers [31] that frequently elevate the head from the ground, this bright chin coloration is positioned by these snakes where it could serve as a visual signal for mate selection. Therefore, Eastern Indigo Snakes might exhibit sexual dichromatism. Alternatively, color might vary among individuals because it is controlled by genes that are under natural selection or neutral evolution. As discussed above, this species occupies varying habitats at different times of the year, and, therefore, any signals associated with the bright chin colors would have to operate across these divergent backgrounds. We tested these alternative hypotheses by examining whether coloration of the sublabial, submaxillary, and ventral scales of this species differed by sex (expected under sexual selection or if natural selection differs between males and females, see examples above) or among clutches (expected under stabilizing selection, neutral evolution, or maternal effects).

## Methods and Materials

### Collection and Care

We obtained 17 clutches from gravid, free-ranging females that were captured in Georgia. All females were captured by hand under Federal Fish and Wildlife Permit #TE32397A-0. These females were then transported to and maintained at Auburn University until the snakes deposited eggs (Table 1). Due to conservation concerns adult females were immediately returned to their point of capture. Eggs were incubated at a constant temperature (26°C) on a moistened cloth to maintain high humidity. Hatchlings emerged during August 2008 (clutches A, B, and C), July and August 2009 (clutches D, E, F, G, H, I, and K), and August and September 2010 (clutches L, N, O, P, Q, R, and S). These snakes were reared at Auburn University for up to one year. All color measurements (see details below) were recorded within six months of hatching. Each individual, regardless of clutch or sex, consumed a similar diet (including quantity) of fish, frogs, lizards, mice, and chicks. Sex of individuals was determined via use of a sterile probe when individuals were at least one year of age. After the conclusion of our data collection, snakes were maintained at Auburn University until either (1) their release in the Conecuh National Forest (31.11 N 86.54 W) as part of a repatriation project or (2) their donation to a breeding program associated with the repatriation project. The larger, repatriation project was designed in conjunction with Auburn University, the Orianne Society, Alabama Department of Natural Resources, and Zoo Atlanta. In the rare case that a snake died before release (n = 5), it was donated to the Auburn Natural History Museum of Herpetology.

**Table 1:** Sample sizes of females and males per clutch.

Clutch	Date of egg collection	*N_females_*	*N_males_*
A	February 2008	5	3
B	February 2008	3	5
C	March 2008	3	1
D	January 2009	4	3
E	January 2009	1	1
F	January 2009	2	8
G	January 2009	3	5
H	January 2009	0	5
I	January 2009	0	4
K	January 2009	0	7
L	February 2010	3	4
N	February 2010	2	4
O	March 2010	2	7
P	March 2010	5	4
Q	March 2010	3	1
R	March 2010	4	2
S	March 2010	7	1
Total		47	65

This study was carried out in accordance with the recommendations in the Guide for the Care and Use of Laboratory Animals of the National Institutes of Health and the Guidelines for Use of Live Amphibians and Reptiles in Field and Laboratory Research (2^nd^ edition) of the American Society of Ichthyologists and Herpetologists. The protocol was approved by the Committee on the Ethics of Animal Experiments of Auburn University (PRN 2007-1142 and 2010-1750).

### Spectrometry

**Figure 2:**
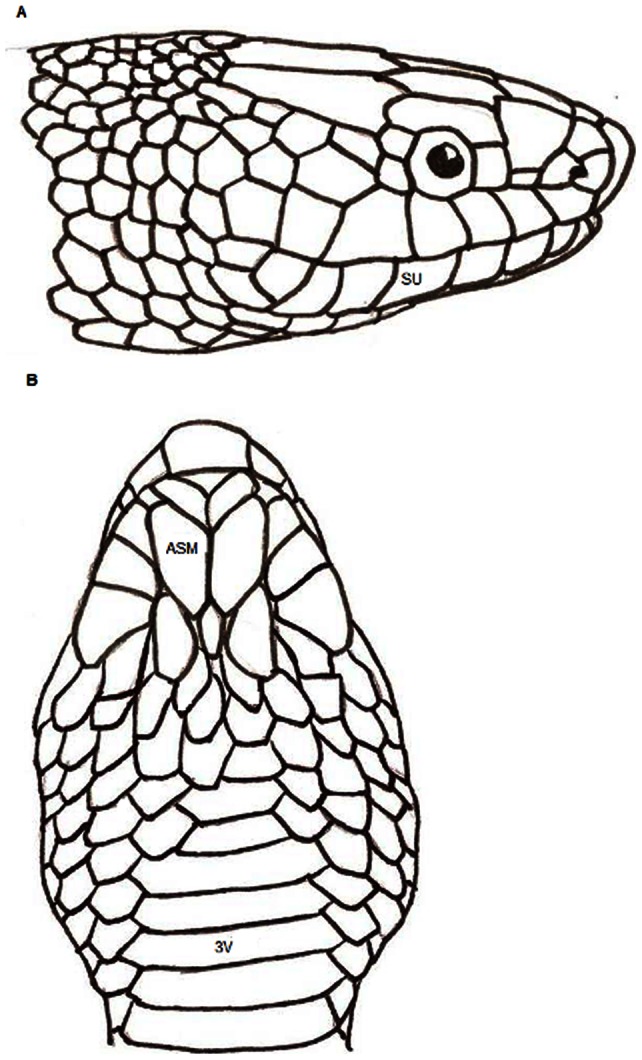
Diagram showing which scales were used to collect color spectrophotometry reflectance values. a) Diagram of the right lateral head showing the fourth sublabial scale (SU). b) Diagram of the ventral view of the head and neck showing the anterior submaxillary (ASM) and third ventral (3V) scales.

Spectrometry measurements were used to characterize important aspects of color and data collection was conducted following the completion of a shed cycle by each snake. We measured scale coloration of individuals of Eastern Indigo Snakes using an Ocean Optics S2000 portable spectrometer (range 250–880 nm: Dunedin, Florida) coupled to a PX-2 Pulsed Xenon light source. We placed a bifurcated fiber-optic cable mounted in a metal probe at an angle of 90° to the plane of the measured scale surface. Before measurements were recorded, we used a white standard to calibrate reflectance measurements (with the white standard set as 100% reflectance). Because color variation in these snakes occurs on the lateral surfaces of the head, the chin, and the first several ventral scales, we measured color from one scale in each of these regions to compare color between sexes and among clutches (Fig. 2): one scale on the right side of the head (the fourth sublabial), one scale on the right ventral surface of the head (anterior submaxillary), and the center of one ventral scale (third ventral). For each scale, we recorded three measurements from the surface of the scale, and these measurements were averaged to obtain a mean spectrometry reading for each scale on an individual. Spectral data were gathered as percent reflectance (of the white standard) at 1 nm wavelength increments from 300–700 nm.

### Statistical Analyses

We compared color between sexes and among clutches using two methods. The first method examined overall color differences across the entire spectrum (300–700 nm); the second method tested differences within the UV, yellow, and red colorbands. Each methods reveals different aspects of color [32]. The first method surveys for differences anywhere in the spectrum, while the second method tests for differences in portions of the spectrum specified a priori as being biologically meaningful. Use of both methods assures that unexpected differences might be revealed while simultaneously focusing on expected differences; therefore, we have included both methods as suggested by Montgomerie [33]. Each of these methods is described in more detail below.

To determine if the entire spectrum of color differed between sexes and among clutches, we first performed a Principle Components Analysis (PCA), using the covariance matrix, to reduce the number of dimensions of color from 401 (representing 300–700 nm) to the number of PC axes that contained variation (PC axes that contained no variation were discarded). Using the resulting PC scores, we performed a two-factor multivariate analysis of variance (MANOVA) with sex and clutch as the factors and included an interaction term between the factors. If the interaction term was not significant, we removed this term and repeated the analysis. If color differed among clutches, we performed all possible pair-wise comparisons among clutches. If the interaction term was significant, we performed pair-wise comparisons between males and females within each clutch. For these pair-wise comparisons, as well as those below, we used sequential Bonferroni correction for multiple comparisons.

While there are various methods to analyze color data [33], using the methods above are most appropriate in this study. To perform statistical analyses, the number of variables in the original dataset (401) is too great with our sample size; therefore, a method to reduce these variables is necessary (PCA). One alternative to the above is to use PC1 to approximate brightness [33]. PC1 often has high, positive loadings for the original raw reflectance scores and is often highly correlated with total brightness [34]. However, PC1 is not perfectly correlated with brightness and other PC axes are not independent of brightness. In addition, we were interested in the complete variable of “color,” not just brightness; therefore, we included all PC axes.

For the second method, we used segment classification (described in [33]) by first creating categories of reflectance values for ultraviolet (UV; 300–400 nm), yellow (551–625 nm), and red (626–700 nm) colorbands. For studies of color, in general, reflectance is higher at some wavelengths than at others within the visible spectrum, and the wavelengths that are higher are perceived by an observer. This is referred to as “hue” and includes the words typically used to describe colors (red, yellow, etc.). Colors for these wavelengths are, thus, “expressed” by an individual. Yellow and red colorbands were included because these are the hues expressed by these variable scales (Fig. 1). We included UV because the human eye cannot detect these wavelengths and many other species display variation in the UV spectrum (see [5]); therefore, making this a potentially important color component when examining dichromatism. We did not include violet, blue, or green colorbands (401–550 nm) in our analyses as these hues are not expressed in these scales by Eastern Indigo Snakes. This reasoning follows that of Siefferman and Hill [35]–[37]. For each colorband, we calculated brightness and chroma (also known as saturation) using formulas described in Montgomerie [33]. Brightness is the amount of light coming from a surface, and brightness of each colorband was calculated as the percent total reflectance of that colorband. Chroma is the degree to which a color is “pure” (i.e. composed of a defined set of wavelengths to the exclusion of other wavelengths) and was calculated as the proportion of light in these colorbands relative to total reflectance across the entire spectrum (300–700 nm). Chroma can be interpreted as the amount of light reflected in a specific colorband in comparison to the amount of light reflected in all other colorbands. Then, we performed two-factor analysis of variance (ANOVA) using clutch and sex as the factors (as above, we retained the interaction term between these factors if it was significant) to determine which color measurements (brightness and chroma for UV, yellow, and red) were significantly different. We did not perform pair-wise comparisons because we felt that correction methods would not be sufficient to account for possible Type I errors as this would require 2754 comparisons (153 clutch comparisons for 3 scales and 6 colorband components per scale).

## Results

**Figure 3:**
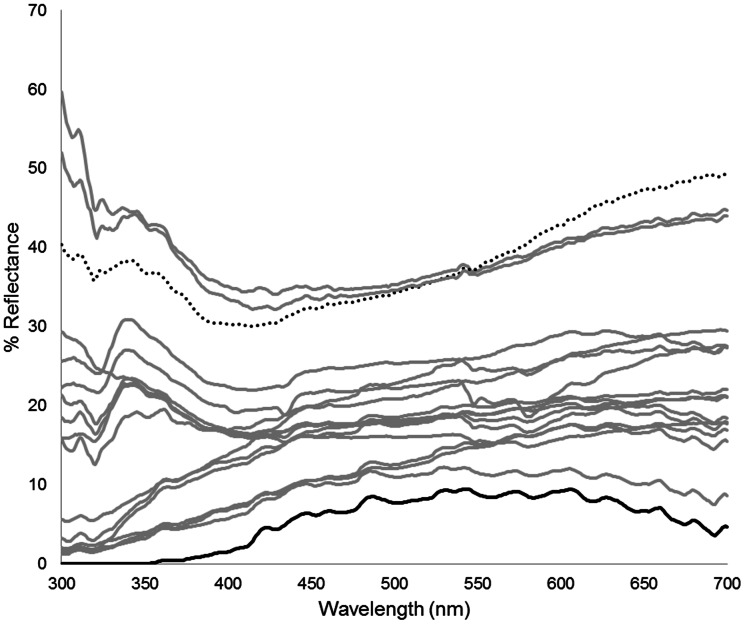
Mean spectral reflectance curves for the sublabial scale. Each line represents a clutch. Clutches B (dotted black line) and R (solid black line) were significantly different (*p* = 0.0003) and were given a unique color pattern to stress this difference. All other clutches were not significantly different and were represented by gray lines.

We tested whether clutches and sexes differed in reflectance of the visible and UV spectra (300–700 nm) on three scales: the fourth sublabial, anterior submaxillary, and third ventral. PCA reduced these data to 18 (sublabial scale) or 19 (submaxillary and ventral scales) axes summarizing variation across these wavelengths in the form of 18 (sublabial) and 19 (submaxillary and ventral) PC scores for each individual. For all three scales, MANOVA of these scores yielded an interaction term of clutch by sex that was not significant and, therefore, it was removed from the analyses (results not shown). We found that the sublabial scale differed in color among clutches (*F_288,968_*  = 2.99; *p*<0.0001; Fig. 3), but not between sexes (*F_18,77_*  = 0.90; *p* = 0.58). For the sublabial scale, clutches B and R were the only ones that were significantly different from each other (*p* = 0.0003; Fig. 3). Color of the submaxillary scale differed among clutches (*F_304,980_*  = 2.74; *p*<0.0001), but not between sexes (*F_19,76_*  = 1.10; *p* = 0.36). For the ventral scale, color differed among clutches (*F_304,980_*  = 2.66; *p*<0.0001) but not between sexes (*F_19,76_*  = 1.23; *p* = 0.26). No two clutches differed when pair-wise comparisons were performed on data for submaxillary and ventral scales.

**Figure 4:**
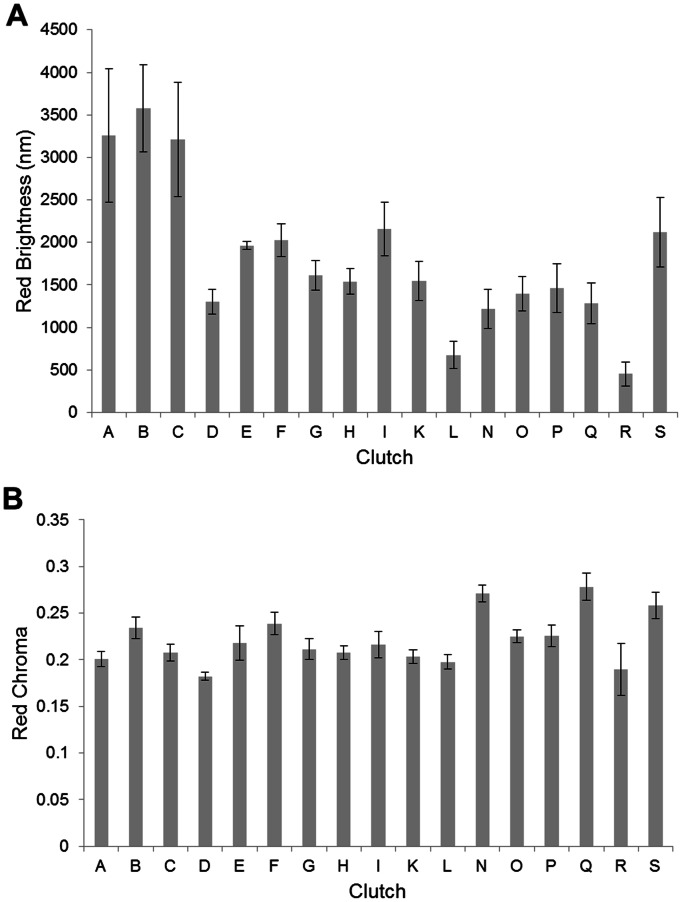
Comparison among clutches in the sublabial scale for a) red brightness and b) red chroma. Error bars represent standard errors.

When we examined brightness and chroma of UV, yellow, and red colorbands for all three scales, the interaction terms for seven of the nine comparisons were not significant and were removed from those analyses; for the ventral scale, the interaction was significant in analyses of red and yellow chroma and was retained in the analysis (discussed further below). For all three scales, UV brightness, red brightness, yellow brightness, UV chroma, yellow chroma, and red chroma differed among clutches but not between sexes (Table 2). We included only the results for the sublabial scale in red brightness and red chroma (Fig. 4) to demonstrate the variability in red color among clutches.

**Table 2:** Summary of results examining differences in colorband components among clutches and between sexes of the sublabial, submaxillary and ventral scales.

Sublabial			F^†^	P	
	UVB	Clutch	8.76	<0.0001	*
		Sex	0.15	0.7	
	YB	Clutch	4.71	<0.0001	*
		Sex	0.42	0.52	
	RB	Clutch	5.50	<0.0001	*
		Sex	0.79	0.38	
	UVC	Clutch	17.67	<0.0001	*
		Sex	0.072	0.79	
	YC	Clutch	13.19	<0.0001	*
		Sex	0.061	0.81	
	RC	Clutch	4.64	<0.0001	*
		Sex	2.18	0.14	

Submaxillary					
	UVB	Clutch	4.13	<0.0001	*
		Sex	0.18	0.67	
	YB	Clutch	2.40	0.0047	*
		Sex	0.045	0.83	
	RB	Clutch	2.53	0.0029	*
		Sex	0.052	0.82	
	UVC	Clutch	12.51	<0.0001	*
		Sex	0.042	0.83	
	YC	Clutch	9.05	<0.0001	*
		Sex	0.28	0.60	
	RC	Clutch	2.70	0.0015	*
		Sex	0.71	0.40	

Ventral					
	UVB	Clutch	6.24	<0.0001	*
		Sex	0.0013	0.97	
	YB	Clutch	2.09	0.015	*
		Sex	1.10	0.30	
	RB	Clutch	2.40	0.0047	*
		Sex	1.34	0.25	
	UVC	Clutch	13.73	<0.0001	*
		Sex	0.10	0.75	
	YC	Clutch	10.42	<0.0001	*
		Sex	0	1.00	
		Clutch by Sex	1.86	0.047	*
	RC	Clutch	8.55	<0.0001	*
		Sex	0.12	0.74	
		Clutch by Sex	3.69	0.00014	*

UV represents ultraviolet (300–400 nm). Y represents yellow (551–625 nm). R represents red (626–700 nm). B represents brightness. C represents chroma. ^†^Degrees of freedom for all tests without the interaction term are 16 for clutch, 1 for sex, and 94 for residual; for tests with the interaction term, residual dg is 81. * Indicates significance.

## Discussion

Our direct hypotheses were to test if sexual dichromatism occurs or if color variation is related to clutch in Eastern Indigo Snakes. In this study we found that this species does not exhibit sexual dichromatism, but that coloration does vary among clutches. Thus, we reject both contradicting statements previously postulated about color in Eastern Indigo Snakes: Mount's [29] suggestion that red coloration signals that an individual is female and Moulis' [30] suggestion that bright-chinned individuals are males. Because color is not associated with sex, we have no evidence that sexual selection plays a role in the evolution and maintenance of color variation in Eastern Indigo Snakes the way it does in sexually dichromatic iguanians [23], [21], [24], [20], [22]. Sexual selection was not directly measured in this study and, therefore, cannot be ruled out as the mechanism for maintaining the color variation described here; however, for sexual selection to play a role in this system, the same traits for both males and females would need to be preferentially selected. Therefore, color may be maintained by sexual selection through some alternative mechanism. As only one example, color in females could be a byproduct of selection for color in males if it is inherited via autosomal chromosomes.

Instead, color is a polymorphic trait that (1) may be influenced by some other type of selection (for example, predator avoidance via crypsis) or (2) may evolve via random processes such as drift. The association between color and clutch leads us to conclude that color most likely results from a genetic mechanism (unrelated to sex) or from maternal effects (i.e. inputs from the mother during egg production). Although our measurements are for first-year snakes and color may change as an individual ages [38], we have followed these same snakes through their first several years of life as part of a repatriation project and have observed no obvious changes in color within these individuals. Similarly, coloration could be associated with variable rearing techniques, but we feel that this alternative is unlikely as all eggs and hatchling snakes used in this study were reared under controlled temperature, humidity, light, and feeding conditions.

When trying to determine if color differences are used by interacting individuals, it is important to understand the spectral sensitivity of those individuals [39]. Unfortunately, spectral sensitivity curves are not known for Eastern Indigo Snakes and can only be inferred for other snakes in general [28]. In our case, inclusion of spectral sensitivity curves is unnecessary. In general, quantitative analyses, such as those presented here, are more sensitive than vision and are more likely to detect differences than an observer would. Since color is statistically invariant between sexes, spectral sensitivity curves would not change this result. Sensitivity curves would likely demonstrate that individuals would not be able to detect the statistical differences between clutches, but we do not claim that individuals belonging to different clutches could be distinguished by these snakes.

Many species display color polymorphisms that are genetically inherited. For example, the melanocortin-1 receptor (MC1R) gene is responsible for the level of melanism (production of melanin, the pigment that influences black, brown, and russet colors) in a variety of vertebrate species [40]–[43]. Furthermore, the inheritance patterns of color polymorphisms in snakes [44]–[46] and other vertebrates [47], [41], [48] are known. Most of these studies examine color polymorphisms related to melanin production, and these different color patterns generally act to decrease detection by predators in varying habitats. We predict that the black color of sublabial, submaxillary, or ventral scales results from increased expression of eumelanin production in these scales, a process that may mask other pigments that may be present. Our conclusion that the degree of melanism differs among clutches is consistent with melanin production undergoing genetic regulation.

Similarly, we infer that the cream-to-red color variation in Eastern Indigo Snakes presented here is influenced by the differential production of red pigments in these scales. Carotenoid and pteridine pigments are known from squamates [49]–[52] and produce red coloration when pigments are present and white or cream coloration when they are not. Red can be produced by one or the other of these pigment types [49], [52], or both pigment types may be present [50], [51]. The production of pteridine pigments is likely activated during embryonic and fetal development as part of the pteridine biosynthetic pathway [53], [52]. Therefore, if the red coloration in these snakes is pterin-based, it is likely genetically inherited. Unfortunately, the color of the chin of the mothers was not recorded and so we do not know whether clutches with red offspring tended to be produced by mothers with red chins. Future studies to answer this question could focus on obtaining hereditary measures of color between parents and offspring.

Another potential mechanism for transferring color from mothers to offspring is through maternal effects. Maternal effects, or non-genetic characteristics of mothers, often contribute to the phenotypic characteristics of a female's offspring. In particular, offspring size and developmental rate are often tied to nutrient allocation to eggs or embryos as well as the environment in which offspring undergo early development [4]. While some red and orange coloration created by carotenoids can be obtained via the diet of squamates, other sources of these colors (e.g. pterins) are not [54]. In species with carotenoids, these pigments may be obtained through the mother, via her diet, during egg production [55], [4], [54], and allocation of carotenoids to eggs may be associated with offspring survival and attractiveness as adults [4]. Therefore, some of the color variation that we observed in *D. couperi* could be influenced by maternal effects.

We are aware of only a few studies that have quantitatively demonstrated sexual dichromatism in snakes, all of which hypothesized that color variation is explained by natural selection working within one sex (e.g., increased crypsis for zigzag male vipers [12], [56], and increased gestational efficiency for darker female Cottonmouths [14]). In addition to these studies, other reports of sexual dichromatism in snakes occur, but these reports are not quantitative, the differences are subtle, and evolutionary explanations for these differences, if any, are not well supported [11]. Thus, snakes (including Eastern Indigo Snakes) appear to have evolved along a highly conserved trajectory on which color and color pattern are consistent between sexes, making both either cryptic or aposematic. This uniformity of color pattern contrasts with the sexually-selected, dichromatic color signaling commonly exhibited between the sexes of the limbed ancestors of snakes. We infer this contrast to be associated with the limited visual system of the burrowing or aquatic ancestor of snakes [57], [58]. In the specific case of Eastern Indigo Snakes, no photoreceptor may be present to allow vision in the red portion of the spectrum [28]; and, in the general case of snakes, there may not be sufficient color vision in females to generate sexual selection based on color patterns in males.

We began this study expecting to support sexual dichromatism as the explanation for color variation in Eastern Indigo Snakes, because this hypothesis had emerged from natural historians experienced with the species. Our data clearly reject this hypothesis and our discussion above explains why we now think sexual dichromatism in snakes is expected to be rare. But, the variation in color within Eastern Indigo Snakes is striking to the human eye, and, therefore, seems to demand some explanation. Of course, color variation may emerge from the action of neutral alleles that have not had time to drift to fixation. Our gravid females were collected from the largest remaining populations in areas of southern Georgia where habitat quality has remained similar to the presumed ancestral landscape because of persistent management with fire administered across broad landscapes. We would expect adequate time for fixation in such habitats. If color variation results from some process other than drift, then the variation that we documented could serve as a badge signaling offspring quality, perhaps determined by maternal effects. We offer this as the next hypothesis to be considered.
